# Exploring determinants of hand hygiene among hospital nurses: a qualitative study

**DOI:** 10.1186/s12912-020-00505-y

**Published:** 2020-11-25

**Authors:** Mohtasham Ghaffari, Sakineh Rakhshanderou, Ali Safari-Moradabadi, Hassan Barkati

**Affiliations:** 1 Department of public health, School of Public Health and Safety, Shahid Beheshti University of Medical Sciences, Tehran, Iran; 2 Student Research Committee, Shahid Beheshti University of Medical Sciences, Tehran, Iran; 3 Health Education and Health Promotion, Social Deputy, Tehran University of Medical Sciences, Tehran, Iran

**Keywords:** Hand hygiene, Hand washing, Theory of planned behavior, Nursing

## Abstract

**Background:**

The present research is a qualitative one aiming to determine factors affecting hand-hygiene behavior of the nursing staff in Shariati Hospital of Tehran, Iran**.**

**Methods:**

This was a qualitative study performed using content analysis approach. Considering the aim of the study, 16 in-depth semi-structured interviews were held with the nursing staff of Shariati Hospital of Tehran University of Medical Sciences. A convenient sampling was performed and continued until data saturation and until no new codes and categories were obtained. Data were analyzed through a qualitative content analysis based on the Graham and landsman method. Directed qualitative content analysis was done in order to analyze the data.

**Results:**

The results of this study revealed 3 main themes in the Theory of Planned Behavior (TPB) (attitude, subjective norms and perceived behavioral control) and 8 main themes in the outside the framework (environment, perceptions, life style, morality, education, organizational culture, salience and personality).

**Conclusion:**

Due to the other factors also found in this study, an integration of theories and models for designing of interventions is recommended to increase adherence to hand hygiene behavior.

**Supplementary Information:**

The online version contains supplementary material available at 10.1186/s12912-020-00505-y.

## Background

Hand Hygiene **[**HH] is a simple way to reduce hospital infections, to prevent the spread of antimicrobial resistance and to increase patients’ security [1]. A mere hand washes stands as a primary way of preventing the spread of hospital infections. A hand wash would cut down on 50% of hospital infections [2]. Hospital infections lengthen patients’ stay in hospital, impose high costs on patients and the health system of the whole country. They may also cause mortalities [3]. According to the results of a review study, Compliance with HH Guidelines were lower in intensive care units (30–40%) than in general ward (50–60%), lower among physicians (32%) than among nurses (48%), and before (21%) rather than after (47%) patient contact. Also, compliance rates higher than 50% were found among 41% of samples of nurses, 20% of physicians, 47% of other health care worker(s), and 25% of health care worker(s) of unreported profession [3]. Although the rising trend of HH protocols is indicative of the lower occurrence of infection, still taking HH serious among other specialized teams has remained poor [4]. According to the figures reported by WHO, 1,400,000 people worldwide suffer from the side effects of hospital infections, per second. In developing countries, the rate of preventable infections induced by healthcare services was estimated 40% higher [5]. Hospital infections are recognized among the key risk factors in healthcare centers. HH stands as the first step that needs to be taken to control these infections [6]. Evidence has it that there exists a wide gap between what people know and how they behave in the healthcare system [7]. A myriad of factors can affect the clinical behavior of the health staff, awareness of which can help to modify their behavior [8]. A number of obstacles known to HH are: skin irritation caused by HH factors, limited access to HH facilities, interference with patient and medical staff communications, belief in the priority of patient’s need for HH, wearing gloves, absentmindedness, unawareness of instructions, inadequate time for HH, work overload, insufficient personnel, lack of scientific information about the definite correlation of HH and hospital infections. Some of these obstacles to HH have been evaluated in descriptive studies or have turned into quantitative results. Unawareness of HH guidelines, unawareness of emergency cases of HH upon providing a patient with healthcare services and unawareness of the risk of transmitting infectious factors are the main obstacles to HH. Moreover, a number of the medical staff believed that they would wash their hands when necessary, but on examination it became clear that they were not doing so [6].

Some studies show that various factors such as knowledge, beliefs and attitudes can affect the HH compliance rates [9–11]. Also, based on the self-report results presented in various studies, the factors related to poor hand washing are: (1) hand washing causes irritation and dryness, (2) lack of soap and paper towels (3) congestion and time Insufficient; (4) underemployment / overcrowding; (6) Priority of patients’ needs and (7) Low risk of infection from patients [12–14].

Following a literature review, we determined that no qualitative study regarding nurse HH (Specifically) has been performed in Iran. Therefore, the objective of this study was to assess various aspects of HH from the perspective of nurse in Iran.

## Methods

As a qualitative study, the present research aimed to determine factors affecting HH behavior of the nursing staff in hospitals affiliated with Tehran University of Medical Sciences.

### Participants & study setting

The nursing staff of Shariati Hospital of Tehran University of Medical Sciences was included in the convenience method. Participants entered the study from different sections of the hospital, such as the CCU, NICU, ICU and Emergency. Total number of contributions the participants was 16 in the study. Convenient sampling technique was used in this study. The inclusion criterion was voluntary participation.

### Structured-interview guide

Semi-structured individual interview were used to collect data. Since this type of interview is profound and flexible, it fits in well with qualitative research [15]. In advance to the interviews, the purpose of the research, participants’ rights and the need for voice recording were made known to all subjects. Participants were provided information about the study and verbal consented by proceeding to take the survey. The key questions used as interview guideline were designed based on TPB (Table 1).

**Table 1:** Topics covered to lead interviews

Topic	Some discussion point
Attitudes	1. How do you define hand hygiene?
	2. How do you feel about hand hygiene?
	3. Why do you think some staff frequently wash their hands and keep them clean while others do not?
Subjective norms	4. How does the environment and the people around affect hand hygiene?
Perceived behavioral control	5. How can some people adhere to hand hygiene in almost all circumstances?

The accuracy of the research was ensured using Lincoln and Guba criteria [16]: items such as close interaction with participants and review of transcription by check Member to determine the acceptability; researcher neutrality, agreement of members (Peer check) on the interview and codes, classification of similar codes and category in order to Conformability; transcription as soon as possible, independent reading of interview texts and codes by external check, re-reading of all data to confirm the findings (Dependability); fittingness of data, interviewing with different participants (Maximum variation of sampling) and providing direct quotes and examples and rich explanation of data for transferability were possible.

### Face-to-face interviews

The interviews took place in one of the halls of Shariati Hospital, which had all the conditions for conducting the interview. All interviews were conducted individually in a convenient and private context. Each lasted for 25–35 min and interviews continued in number so long as the data were saturated. So that there were no new codes in the last three interviews.

The total number of participants in this study was 16 nurses. As agreed by all participants, all interviews were voice-recorded. Word by word transcription was done to prepare the data for analysis. In a qualitative investigation, the researcher is supposed to sink into data (11). Therefore, the researcher reviewed interview texts several times.

### Ethical considerations

Among ethical considerations of this study were subjects’ informed consent, anonymity and confidentiality of data during analysis. A few subjects did not consent to the voice recording, which was replaced by a mere real-time manual note-taking. This article has been extracted from a PhD thesis on Health Education and Health Promotion that has been confirmed in ethics committee. Also, it has been approved and supported as a research project by Environmental and Occupational Hazards Control Research Center in Shahid Beheshti University of Medical Sciences, Tehran, Iran (SBMU.RAM.REC.1394.540).

### Analysis

Directed qualitative content analysis was done in order to analyze the data. This method is used so as to analyze the texts. In this method, through a systematic classification procedure, codes and themes are identified. Content analysis is beyond the mere extraction of actual content from textual data. It helps to find hidden themes and patterns in the text produced by participants [17]. The right method to analyze texts qualitatively is guided or theory-based qualitative content analysis. This method was first proposed by Shannon and Hsieh (2005). In guided qualitative content analysis, the initial coding starts with a theory or findings from a similar research. This analysis aims to validate or extend a conceptual framework or a previous theory. The theory selected in the present research can help to concentrate on the research question.

Therefore, in this research, the data were analyzed through six stages including: 1. Researcher’s familiarity with data 2. Extraction of initial codes from data 3. Searching for themes via a review of the extracted codes from the previous stage 4. Review of themes and re-comparing them with data to make sure of their accuracy 5. Defining and entitling themes, and 6. Preparing a final report [18].

## Results

It should be noted that the categories obtained from the interview (both in the TPB Constructs section and in the out of TPB framework section) are all extracted from multiple codes and quotations and have an acceptable frequency due to data saturation. This article briefly mentions a few examples of quotes.

A total number of 16 participants were included in the study. Their average age was 34 years (min = 23, max = 47) and their average work experience was 11.34 years (min = 1, max = 25) (Table 2).

**Table 2:** Participant’s profile

Participant	1	2	3	4	5	6	7	8	9	10	11	12	13	14	15	16
**Gender**^a^	F	F	F	F	F	M	F	F	F	M	F	F	F	F	F	M
**Age**	36	39	40	23	26	26	39	47	42	35	24	29	42	24	39	33
**Work experience**	8	15	21	1	6	6	15	25	20	10	3	6	15	1.5	15	14
**Professional Background**^b^	N	N	HN	N	N	N	N	N	N	N	N	N	N	N	HN	HN

^a^*M* Male, *F* Female

^b^*N* Nurse, *HN* Head nurse

Factors affecting HH extracted from the texts belonged to either TPB framework or outside this framework:

### The main categories related to TPB framework (Table 3)

#### (A) Attitude (include behavioral beliefs and evaluation of outcomes)

In this structure, phrases were extracted such as: The belief to the behavior, Good feeling and satisfaction after hand washing (Behavioral beliefs), Valuing for self-health, the importance of family health (Evaluation of behavioral outcomes).

**Table 3:** TPB constructs based determinants of hand hygiene

The main categories	Sub-categories	Initial codes
**Attitude**: overall sense of like or dislike of a behavior	Behavioral beliefs	▪ The belief to the behavior ▪ Good feeling and satisfaction after hand washing ▪ To prevent from infection transmission ▪ To Reduce the use of antibiotics and drug costs ▪ To reduce costs and increase hospital income ▪ Safety of employees ▪ Residence and hospitalization of patient in hospital ▪ To control drug-resistant infections
	Evaluation of behavioral outcomes	▪ Valuing for self-health ▪ Valuing for the health of others ▪ The importance of family health
**Subjective Norms:** have to do with the most important people in one’s life who believe one must or must not show a certain behavior	Normative beliefs	▪ Supervisor’s emphasizes to Hand washing ▪ The effect of Doctor’s believes to Hand washing ▪ Hand washing must begin from the top (management level) ▪ The influence of others in hospital
	Motivation to compliance	▪ Hand washing must begin from the top (management level) ▪ The influence of others in hospital
	Descriptive norms	▪ Effect of behavior by physicians
**Perceived Behavioral Control:** people’s perceptions of their ability to perform a given behavior	Control beliefs	▪ Negligence and laziness ▪ Crowded wards and high workload situations ▪ The impact of emergency situations ▪ Insufficient number of personnel ▪ Lack of concentration due to lack of time
	Perceived power	▪ Possibility of adherence at the crowded situation ▪ Possibility of adherence in every circumstances

**Quotation:** “There was another human being I cared about. For the respect I pay health of him and myself, I do not wish to transmit infections to him or get them from him” (P#6; Par#2).

#### (B) Subjective norms (include: normative beliefs, motivation to compliance, descriptive norms)

In this structure, phrases were extracted such as: Supervisor’s emphasizes to Hand washing, the effect of doctor’s believe to hand washing (Normative beliefs), Hand washing must begin from the top (Motivation to compliance) and Effect of behavior by physicians (Descriptive norms).

**Quotations:**

- **Normative beliefs:** “They are really effective. Those above us set a model for us. When for example I see that the head nurse washes hands always when visiting a patient, I get impressed” (P#1; Par#4).
- **Motivation to compliance**: “others play a key role here, in a work environment such as a hospital, it is seen that some characters are charismatic and the staff tries to act according to their opinion” (P#16; Par#1).
- **Descriptive norms**: As an instance, a participant admitted: “when my top rank does a certain thing, I do too. Also, if I think that the head nurse, although she recommends hand hygiene but does not practice this behavior herself, this behavior of her will have a deterrent effect for me and others like me” (P#2; Par#5).

#### (C) Perceived behavioral control (include control beliefs and perceived power)

In this structure, phrases were extracted such as: Negligence and laziness, Crowded wards and high workload situations (Control beliefs), Possibility of adherence in every circumstances (Perceived power).

**Quotations:**

- **Control beliefs (**Head nurse: “well, when he is pressed for time, there is no way to concentrate, no way to wash hands” (P#3; Par#2).
- **Perceived power:** “being busy should not be an excuse for not washing our hands” (P#1; Par#5).

### The main categories related to outside TPB framework (Table 4)

#### (A) Environment

Refers to the physical and social environment surrounding an individual.

1. **Monitoring and supervision:** some initial code this subgroup Includes: rules and regulations, consistent controls, direct and indirect monitoring.

**Table 4:** Some Other Determinants of Hand Hygiene (Out of TPB Framework)

The main categories	Sub-categories	Initial codes
Environment	Supervision &Monitoring	▪ Law ▪ Continuous controls ▪ Cultivation of hands and reflection to personnel ▪ The institutionalization of monitoring ▪ Supervision and monitoring ▪ Pressure ▪ Obligation Service personnel to hand hygiene
	Reinforcement	▪ System of rewards and punishments to motivate ▪ Encourage writing ▪ Verbal encouragement
	Cues to Action	▪ Poster in sight ▪ Reminders ▪ More advertising (LSD)
	Availability & Accessibility	▪ Availability of sink ▪ Qualities and aromatic liquid soap and Hand rub and paper ▪ Appropriate dryer
	Preferences	▪ Qualities and aromatic liquid soap and Hand rub and paper
	Modeling & Observational Learning	▪ According to the head nurse and professor at hand washing ▪ Benchmarking and being model for others
Perceptions	Outcome expectation**s**.	▪ Preventing from infection. ▪ Residence and hospitalization of patients in hospital ▪ Control treatment resistant infections
	Perceived barriers	▪ Skin dryness (lack of suitable lotions and liquid soaps) ▪ The destruction of Nails (Washing too) ▪ Forget hand washing
Education	Awareness and Education	Patient Education ▪ Training to along patient ▪ Instill the importance of behavior to personnel ▪ Education through the media
Organizational Culture	Organizational climateSense of participation and cooperation	▪ Effect of organizational climate on the individual ▪ Induction of the importance of Hand washing for staff ▪ Patient participation in hand hygiene programs ▪ Culture of hand hygiene (Organization)
Salience	The importance of behavior	▪ Insensitivity to the issue ▪ Mental priorities ▪ Failure to understand the importance of hand hygiene
Life Style	Habit	▪ Initial training of family ▪ Make a habit of washing hands ▪ Culture of hand hygiene (family and community)
Personality	Personality characteristics	▪ Regulatory and being obsessive ▪ Having a strong character person
Morality	Conscience and ethics	▪ Conscience and morality ▪ The responsibility for others and humankind ▪ Neglect and indifference

**Quotation**: “monitoring has a great effect. Hand culture test done a few years ago was very effective indeed. But it no longer exists. They used to let us know of the culture test result” (P#12; Par#7).

2. **Reinforcement:** The initial code, such as encourage writing, seen important, was placed on this sub-category.
  **Quotation**: “we learned through the system to expect positive/negative reinforcement. There should be a difference between who abides by the rules and who does not” (P#15; Par#5).
3. **Cues to Action:** refer to facilitating force which produces a need for doing a certain thing. The initial code, such as poster in sight and reminders was placed on this subscale.
  **Quotations**: “They need to put up advertisements especially LEDs everywhere” (P#8; Par#3), Messages on desktop computers, tracts, and brochures posted in various parts of the hospital can be good reminders of this behavior (P#14; Par#8).
4. **Availability & Accessibility:** system’s commitment to provide the required facilities was among the factors mentioned by the present nurses. Some initial code include: Availability of sink and Appropriate dryer.
  **Quotation**: “The staffs are mostly young females. Regular hand wash irritates their skin. It would be better to provide quality lotions” (P#11; Par#3).
  **Preferences:** among the issues mentioned by the interviewees were quality odorous hand wash liquid and hand-rub which could help promote HH behavior.
  **Quotation**: “The better quality the soap, the less allergic it is and the better stuff it has, the more comfortably the nurses will use it” (P#13; Par#2).
5. **Modeling:** some initial code in this subgroup include: See nurse and head nurse behaviors.
  **Quotation**: “It differs from one unit to another. In a given unit once the head nurse keeps washing hands 5 times, others would see that and follow that” (P#7; Par#6).

#### (B) Perceptions

1. **Outcome expectations:** Refer to belief about the likelihood of the behavior leading to a specific outcome. Participants pointed to prevention of infection transmission, patient’s longer stay in hospital and control of infections resistant to treatments.
  **Quotation**: “Instead of recovering, his disease gets worse” (P#10; Par#4).
2. **Perceived barrier:** It is refer to an individual’s assessment of the obstacles to behavior change.
  In the present study, these concern skin irritation, lack of proper hand wash soap or lotion, loss of nails (due to excessive hand wash), skin allergy and forgetting to wash hands.
  **Quotation**: “we use too much liquid and our skin gets irritated and this all plays a role”.
  **Education:** Lack of awareness of hand infections, patient education, education through the media, were in this sub-category (P#4; Par#2).
  **Quotation**: “Awareness rising should be through the media” (P#9; Par#5).
3. **Organizational culture:** refers to the main values, assumptions and interpretations of approaches characterizing an organization. Initial codes in the sub-categories included: one’s priority of HH in the public sector as compared to the private sector and conveyance of the importance of work to the personnel.
  **Quotation**: “In private hospitals you do what others often do. The context dominates you and keeps imitating others. There is a fear of conditioning” (P#14; Par#3).
4. **Salience:** The sensitivity of the subject, mental priority and underestimating the role of hygiene were included in this subgroup.
  **Quotation**: “It is very important to see hand washing as a primary priority” (P#15; Par#6).
5. **Personality:** this is Sub-category, initial codes such as: characteristics as being disciplined and having a strong personality were included.
  **Quotation**: “I think they have a strong personality, because they believe where there is a will, there is a way” (P#11; Par#4).
6. **Morality:** It refers to covers human spiritual and natural characteristics. Initial codes were including: conscience, fairness, feeling responsible towards others, indifference and lack of attention.
  **Quotation**: “How do they feel responsible? Two people working side by side in an office, but working differently. It all depends on their conscience and morals” (P#12; Par#7).
7. **Life style:** Refers to the specific way of life led by an individual, group or a society. Initial codes in the Sub-category were: establishment of washing in one’s unconscious mind, family training, establishment of the hand washing habit, creating the culture of hand washing (both in families and society).
  **Quotation**: “Too often we are snowed-under but yet for me, it’s part of my within, my unconscious” (P#5; Par#3).

## Discussion

In the present research, TPB constructs along with environment, perceptions, education, organizational culture, salience, life style, personality and morality were recognized as effective factors of HH. The main categories of TPB were attitude, subjective norms and perceived behavioral control.

### The main categories related to TPB framework

#### Attitude

In the present study, all participants had a positive attitude towards HH. In Barrett’s qualitative research (2008) in the U.K., nursing university students had a positive view towards HH behavior [12]. Other studies that reported positive attitudes were more likely to improve or predict compliance [3, 9, 19, 20]. Studies have found that an HCW’s attitude has little effect on compliance [21–23].

#### Subjective norms

According to the present results, the participants mentioned supervisors’ care about hand-washing, doctor’s belief in hand-washing and peer-effect (in hospital) as the effective factors. These indicators dealt to some extent with abstract norms and in fact revealed nurses’ motivation and obedience of their seniors in hospital. In an investigation conducted by van Beeck et al. (2009) in the ICUs of 5 hospitals, medical students stated directly that they imitated professors’ and higher ranks’ behavior [24]. In their research, Whitby & Perkins maintained that colleagues’ behavior can affect their healthcare behaviors [25, 26]. We can infer that one key predictor of hand-washing behavior among young and inexperienced staff is perceived social pressure by the seniors [24, 27–29]. In some other research, Van Beeck et al. referred to lacking positive social norms and models among physicians and managers as an inhibiting factor of showing hand-washing behavior [24]. Thus, as in social contexts, enjoying appropriate positive models and norms plays a key role. Moreover, according to experience, people tend to imitate managers’ behaviors. Thus, it seems that if the head-nurse, doctor, infection control nurse and other managers show inappropriate hand-wash behavior, it can directly affect the performance of the other personnel.

The present findings managed to extract such meaningful units as “critical and crowded condition of the ward as well as overwork”, “effect of emergent conditions”, “inadequate workforce” and “lack of concentration due to insufficient time”. These units are concerned with the controlling beliefs as in the perceived behavioral control in the planned behavior theory. It in fact points to the perceived power and in the case of showing healthy and hygienic behavior, washing hands is done in all conditions. As perceived by these individuals, this behavior is well established in them.

According to the present findings, the following sub-categories of environmental effects affected the target behavior: supervision and monitoring reinforcement (reward and punishment system for motivation in the form of written appreciation or oral reminding by the colleagues), cue for action (e.g. putting up posters where they can be easily seen), easy access (easy access to sink and a proper drier), preferences (quality and odor of the washing liquid and hand-rub), observational modelling and learning (attention to the head-nurse and professor in the hand-wash process). These indicators obtained from this research help us to conclude that the surrounding environment can be a determining factor in showing healthy behavior. In an investigation entitled as “investigating the effective factors of HH behavior among healthcare providers in ICU: a qualitative study”, Ravaghi et al. (2014) found the following as the main obstacles to performing HH behavior: formal/informal control, improper spatial design, limited facilities and equipment [30]. It seems that thought the institutes have succeeded in motivating and propagating the target behavior, it is essential to monitor the staff strictly so as to promote the staff’s behaviors concerning the hand-wash [31].

Perceptions were among the main themes extracted from this research. Concerning the former, mention was made of the worsening of the disease, preventing infection transmission, lengthening patient’s stay in hospital and controlling infections resistant to treatments. The latter included allergic skin, skin irritation (lack of quality lotion or liquid soap), loss of nails (excessive washing) and forgetting to wash hands. Some other research findings concerning the obstacles to hand-washing were consistent with the present research. They mentioned inadequate time, too many patients cared by a nurse, time-consuming hand-wash, dry and irritable skin caused by recurrent washing, inadequate lavatories in different sections of healthcare centers, inaccessible lavatories, the wrong belief that wearing gloves makes a hand-wash redundant and low awareness of the significance of hand-wash as the main obstacles to show the healthy behavior [12, 32–34]. However, healthcare authorities need to raise others’ awareness and remove the wrong belief that wearing gloves can replace washed hands.

Education and awareness were among the other themes extracted from this research and were comprised of such meaningful units as “unawareness of unclean hands” and “raising personnel’s awareness of the significance of hand-wash”. In order to think of effective policies to promote HH, the current state and staff’s awareness and performance should be examined [3]. In a descriptive research conducted by Nabavi et al. (2013) among a medical staff, awareness level was reported to be moderate and attitude and HH behavior were found to be poor [35]. A wrong belief was also found to be prevalent among the health staff to wear gloves instead of washing hands [24]. This finding is consistent with some other research conducted in ICU that showed though the nursing staff wore gloves in the majority of cases, after taking them off, they hardly ever wash or disinfect their hands [36]. However, there are mixed findings that show either positive or negative effects of wearing gloves on washing hands afterwards [37]. They must have persuaded themselves that wearing gloves is a proper replacement for cleaning hands and protects both the patient and themselves from infection. On the other hand, Parsi et al. do not perceive gloves as a good replacement for clean hands and maintain that gloves are not fully impenetrable [38]. When the healthcare providers do not wash their hands properly before wearing gloves, it seems that why they wear gloves in the first place is more to protect themselves rather than to prevent and control the transmission of diseases to patients. Nevertheless, they need to know that wearing gloves does not suffice on its own to protect them fully from the patient-induced infection [37].

Lifestyle, personality, and organizational culture, as other classes derived from this article, play a key role in hand washing behavior. Family education, workplace rules and regulations, and a culture of hand hygiene can help promote this behavior. On the other hand, the experience of performing the behavior must become a habit, otherwise the intention to perform the action will lose its importance for the person [39–41]. In relation to organizational culture, Larson et al. (2000) stated that efforts to improve HH performance have been insufficient because little attention has been paid to the care system, expectations, and organizational culture [42].

Morality was a theme derived after the analysis of interviewees’ accounts. One’s perceived morality of a behavior as well as perceived feeling of responsibility seriously affect one’s performance of an action or refraining from that. A few participants mentioned HH as a moral norm in preventing the transmission of infections. This norm is a philanthropic reality and can be activated via an individual’s natural attitude. It occurs when one believes that his key moral values have been approved. Moral norms can play a role in staff’s compliance with the rules of infection control. In their research entitled as “different social and moral norms among medical students”, Roberto et al. (2010) concluded that the two variables, mental and moral norms are good predictors of medical students’ compliance with HH instructions [43].

## Conclusion

The present research identified factors affecting HH among these specific nurses who work in these conditions in these specific work-placement. It also determined domains which need to be considered for assessment and intervention by health-care specialists. This research was indicative of a number of characteristics and beliefs relevant to HH behavior, which were related to behavior change theories and models. Therefore, it emphasizes on the importance of using behavior change theories and models in designing interventions with the aim of promoting the performance of HH behavior. However, due to the nature of the qualitative study, further research is needed in the future to explore the relationship between the identified variables, identify other determinants, and generalize the findings. Finally, the present research is a form of shared experience which can contribute to other similar body of research. Therefore, the need for theory-based interventions following an integrated approach comprised of theories and models seems to be a great help. There is a need for further similar research to identify hygiene determinants in the light of other theories and models especially IBM.

## Supplementary Information

**Additional file 1.** Interview guide.

## Data Availability

The datasets used and analyzed during the current study are available from the corresponding author on reasonable request.

## Abbreviations

HH: Hand hygiene
WHO: World Health Organization
Par: Paragraph
