# Supplementary material for: Exploring determinants of hand hygiene among hospital nurses: a qualitative study
Source: BMC Nurs. 2020 Nov 25;19:109. doi: 10.1186/s12912-020-00505-y (PMC7689993; doi:10.1186/s12912-020-00505-y)
Supplement: Supplementary file 1 — Additional file 1. Interview guide. [file 12912_2020_505_MOESM1_ESM.docx]

Additional file: Interview guide

1. How do you define hand hygiene?
2. How do you feel about hand hygiene?
3. Why do you think some staff frequently wash their hands and keep them clean while others do not?
4. How does the environment and the people around affect hand hygiene?
5. How can some people adhere to hand hygiene in almost all circumstances?
